# Complementary and Alternative Therapies for Inflammatory Diseases

**DOI:** 10.1155/2016/8324815

**Published:** 2016-12-25

**Authors:** Xiang Liu, Ying-Ju Lin, Yong Cheng

**Affiliations:** ^1^National Institutes of Health, Bethesda, MD 20892, USA; ^2^China Medical University, Taichung 40402, Taiwan

Many diseases have been demonstrated to associate with acute or chronic inflammation, such as rheumatoid arthritis, Alzheimer's disease, depression, Kawasaki disease, and even cancer. However, treatment options for inflammatory diseases have been limited to anti-inflammatory medications (e.g., acetaminophen) or steroid hormones. Complementary and alternative medicines (CAMs) provide natural and effective protection for those who suffer from inflammatory diseases. This special issue is focused on the evidence-based research of CAMs for treatment of inflammatory diseases.

Inflammation occurs in response to various cellular stresses including infection, irradiation, or chemical or physical injury. One of the major responses during inflammation is the release of cytokines and/or chemokines. Y. Y. Choi et al. showed that Dangguibohyul-Tang (DBT), a herbal formula composed of* Astragalus membranaceus* (AM) and* Angelica sinensis* (AS) at a ratio of 5 : 1, helps relieve the inflammatory response through reducing IgE levels and cytokine levels. S. Park et al. showed that pomegranate peel extract (PPE) could alleviate inflammatory reactions, including production of reactive oxygen species (ROS) and expression and secretion of inflammatory cytokines.

Studies on the signaling pathways provide the anti-inflammatory mechanism of CAMs. M. Gu et al. demonstrated that genistin is cardioprotective due to its antioxidant and anti-inflammatory activities via P2X7/NF-*κ*B pathways. T. Mao et al. showed that Qingchang Wenzhong Decoction (QCWZD) ameliorates dextran sulphate sodium- (DSS-) induced ulcerative colitis (UC) in rats by downregulating the IP10/CXCR3 axis-mediated inflammatory response. D. Wang et al. proved that Yupingfeng Pulvis (HFBP) alleviates the inflammation in the lung tissue of mice by reducing the proportion of Th17 cells and increasing the proportion of Treg cells in bronchoalveolar lavage fluid.

Using human umbilical vein endothelial cells, E. S. Choi et al. showed that Samul-Tang (Si-Wu-Tang, SMT) protects vascular endothelium from inflammation and might be used as a promising vascular protective drug. G. O. L. Carapeba et al. used animal models to evaluate the treatment effect of intra-articular hyaluronic acid in 4 dogs with osteoarthritis associated with hip dysplasia compared to traditional conservative treatment.

Hyeonggaeyeongyo-tang (HYT) is an ancient formula of oriental medicine traditionally used to treat rhinitis; however, clinical evidence has not yet been established. M. Kim et al. showed that HYT improved nasal symptoms and quality of life in patients with allergic rhinitis and nonallergic rhinitis. This is the first clinical study to evaluate the use of HYT to treat patients with rhinitis. X. Lu et al. showed that Sequential treatments with Tongsai and Bufei Yishen Granules during acute exacerbation of chronic obstructive pulmonary disease- (AECOPD-) risk window (RW) periods can reduce inflammatory response and improve pulmonary function and shorten the recovery courses of AEs.

E. S. B. Barroqueiro et al. showed that babassu mesocarp extract (EE) has specific antimicrobial activity in vitro and has an important antiseptic effect in vivo possibly due to the antimicrobial and immunomodulatory activity. N. J. S. Lopez et al. evaluated the extrusion process as an alternative for improving the biological potential of sorghum bran: phenolic compounds and antiradical and anti-inflammatory capacity. The extrusion process increased total phenol content in sorghum bran, which positively affected antiradical capacity.

In this issue, C.-Y. Cheng et al. reviewed the anti-inflammatory effects of traditional Chinese medicines against ischemic injury in in vivo models of cerebral ischemia. TCMs provide neuroprotective effects through downregulating ischemia-induced microglial activation and expression of proinflammatory cytokines, enzymes, and transcription factors.

These studies provide new insights to treat inflammatory diseases and help us understand better about the mechanism and function of CAMs.

*Xiang Liu* *Xiang Liu* *Ying-Ju Lin* *Ying-Ju Lin* *Yong Cheng* *Yong Cheng*

